# Long non-coding RNAs LOC285194, RP11-462C24.1 and Nbla12061 in serum provide a new approach for distinguishing patients with colorectal cancer from healthy controls

**DOI:** 10.18632/oncotarget.12220

**Published:** 2016-09-23

**Authors:** Chuanxi Wang, Jinyu Yu, Yuping Han, Leping Li, Jie Li, Tao Li, Peng Qi

**Affiliations:** ^1^ Department of Oncology, Shandong Provincial Hospital Affiliated to Shandong University, Jinan, Shandong Province, China; ^2^ Department of Emergency, Shandong Provincial Hospital affiliated to Shandong University, Jinan, Shandong Province, China; ^3^ Department of Gastrointestinal Surgery, Shandong Provincial Hospital Affiliated to Shandong University, Jinan, Shandong Province, China; ^4^ Department of Infectious Diseases, Shandong Provincial Hospital Affiliated to Shandong University, Jinan, Shandong Province, China; ^5^ Department of Pathology, Fudan University Shanghai Cancer Center, Fudan, Shanghai, China; ^6^ Department of Oncology, Shanghai Medical College, Fudan University, Fudan, Shanghai, China; ^7^ Institute of Pathology, Fudan University, Fudan, Shanghai, China

**Keywords:** long non-coding RNA, serum, colorectal cancer, biomarker

## Abstract

Colorectal cancer (CRC) is currently the most prevalent malignant cancer worldwide. However, there is a lack of efficient biomarkers for CRC screening. Accumulating evidence reveals that long non-coding RNAs (lncRNAs) detectable in serum are associated with the genesis and development of various types of cancer. Therefore, we examined the diagnostic ability of lncRNAs in blood samples from patients with CRC by evaluating the levels of 17 CRC- or gastrointestinal cancer-related lncRNAs in serum samples from 71 CRC patients and 70 healthy individuals using reverse transcription-quantitative polymerase chain reaction (RT-qPCR). We detected 13 lncRNAs in serum, three of which displayed significantly different levels between CRC patients and healthy controls. A three-lncRNA signature (LOC285194, RP11-462C24.1 and Nbla12061) identified via stepwise regression analysis showed potential as a diagnostic marker for CRC. The area under the receiver operating characteristic curve of this signature for distinguishing CRC patients from healthy individuals was 0.793 (95% CI: 0.709 to 0.861). The diagnostic ability of this marker was much higher than that of conventional blood biomarkers such as carcinoembryonic antigen (CEA), carbohydrate antigen 199 (CA199), carbohydrate antigen 125 (CA125) and carbohydrate antigen 724 (CA724). Combining this novel marker with conventional biomarkers produced even greater diagnostic ability. Furthermore, the levels of the three lncRNAs decreased after the patients underwent surgical resection. The results of this study suggest an additional marker for CRC screening and provide new directions for further investigation.

## INTRODUCTION

Colorectal cancer (CRC), which currently ranks third in cancer morbidity, is the most prevalent malignant cancer worldwide, with more than 1,000,000 new cases annually [1]. Despite great advancements in CRC treatment, improvements in the survival rates of CRC patients have been restricted by the lack of an efficient screening method. This situation warrants exploration for corresponding indexes. Although colonoscopy is currently recommended as a screening method for CRC, this method is not broadly accepted by the population due to its inconvenience. Detection of biomarkers in circulation is a more convenient and economical screening method. There are four tumor biomarkers for CRC that are widely used in clinical settings: carcinoembryonic antigen (CEA), carbohydrate antigen 199 (CA199), carbohydrate antigen 125 (CA125) and carbohydrate antigen 724 (CA724). Nonetheless, the sensitivity and specificity of these biomarkers are unsatisfactory [2, 3]. Therefore, identifying effective biomarkers for screening and therapy is essential for the detection and treatment of CRC.

Non-coding RNAs, which include microRNAs (miRNAs), long non-coding RNAs (lncRNAs) and circular RNAs, have recently attracted broad attention. MiRNAs and lncRNAs have been studied extensively to determine their complex functions and mechanisms. MiRNAs have been confirmed to be present in the circulation of CRC patients [4–6], suggesting that lncRNAs might also serve as circulating markers for CRC diagnosis in clinical practice. LncRNAs comprise a class of transcripts longer than 200 nucleotides with limited or no protein-coding ability. Accumulating evidence has shown that lncRNAs perform critical biological functions, such as regulating tumor cell proliferation, apoptosis and migration [7, 8]. Because most such studies have been performed using tissue samples, we chose to explore the correlation of lncRNAs in the circulation with CRC.

Circulating biomarkers are easily accessible, requiring only non-invasive sampling methods for detection, and the detection of lncRNAs in serum has been studied intensively [9–11]. LncRNAs have been demonstrated to be stably present in serum for an extended period, even when exposed to room temperature incubation, multiple freeze-thaw cycles, low or high pH, or RNase A [12]. Additionally, accumulated circulating lncRNAs have been demonstrated to have diagnostic value, indicating that lncRNAs hold great potential as powerful tumor biomarkers. For instance, HULC in plasma was demonstrated to serve as a promising novel noninvasive biomarker for the diagnosis and/or prognosis of hepatocellular carcinoma (HCC) [13]. Moreover, the lncRNAs RP11–160H22.5, XLOC_014172 and LOC149086 show potential as biomarkers for predicting HCC tumorigenesis, and XLOC_014172 and LOC149086 exhibit potential for predicting HCC metastasis [14]. However, no systematic study of serum lncRNAs in CRC samples has been performed to date.

In this study, we selected 17 candidate CRC- or gastrointestinal cancer-associated lncRNAs (Supplementary Table S1) identified as displaying dysregulated expression in CRC tissue and evaluated their levels in serum. Our first objective was to determine the levels of these lncRNAs in sera from CRC patients and healthy controls. We then constructed a model for CRC diagnosis based on the serum levels of specific lncRNAs in CRC patients and compared this model with the biomarkers currently used for CRC diagnosis. Finally, we investigated the potential relationships between circulating lncRNA levels and the clinical characteristics of CRC patients.

## RESULTS

### Biomarker selection

To explore the potential utility of serum lncRNAs as biomarkers for CRC, we first explored which lncRNAs can be detected in serum by RT-qPCR and show differential serum abundance between pre-operative CRC patients and healthy controls. In the first step, 13 of the 17 examined lncRNAs were detectable in sera from 10 patients and 10 healthy controls (Supplementary Table S1). LncRNAs that could not be detected were excluded from the next step.

**Figure 1:**
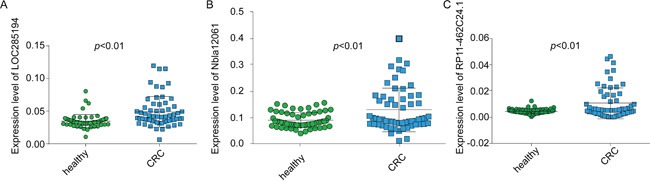
The expression levels of the three lncRNAs in serum Serum levels of LOC285194 **A.** Nbla12061 **B.** and RP11-462C24.1 **C.** were significantly different between CRC patients and healthy subjects, as determined by RT-qPCR; the results were analyzed using a *t*-test.

In the second step, levels of the remaining13 lncRNAs were examined in sera from 30 pre-operative CRC patients and 31 healthy individuals. Five lncRNAs showed significantly differential abundance (up- or down-regulated) between the two groups (Supplementary Table S1). These five lncRNAs and CCAT2 were further evaluated in the third step. Finally, all the data from steps II and III were pooled and analyzed. The results revealed lncRNAs RP11-462C24.1, LOC285194 and Nbla12061 to be significantly up-regulated in serum from CRC patients (Supplementary Table S1, Figure 1A-1C). Therefore, these three lncRNAs were selected as a combined biomarker for CRC and were included in subsequent analyses.

### Diagnostic model construction

**Figure 2:**
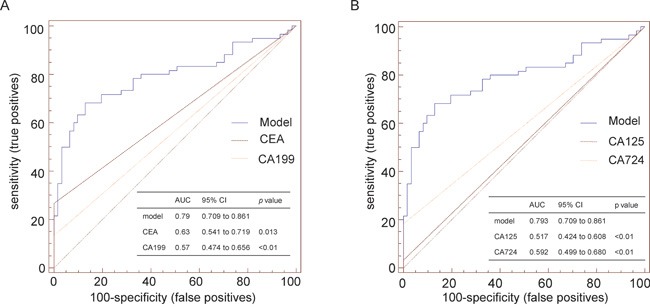
Comparison of the diagnostic ability of a model consisting of the serum levels of three lncRNAsto that of conventional biomarkers **A.** ROC curve was used to discriminate CRC patients from healthy subjects. The AUC represents the diagnostic ability. A. Comparison of the diagnostic ability of a model consisting of the serum levels of the three lncRNAs to that of CEA and CA199. **B.** Comparison of the diagnostic ability of a model consisting of the serum levels of the three lncRNAs to that of CA125 and CA724.

We constructed a diagnostic model using the CRC specimens examined in steps II and III by stepwise analysis of the three above lncRNAs. The AUC of the model combining RP11-462C24.1, LOC285194 and Nbla12061 was 0.793 (95% CI: 0.709 to 0.861), and the sensitivity and specificity of this three-lncRNA serum signature were 68.33% and 86.89%, respectively (Supplementary Table S2 and S3). The levels of clinical biomarkers were assessed in the same serum samples, and the AUC of each biomarker was calculated. We compared the results for the model with those for the clinical biomarkers (CEA, CA199, CA125 and CA724). The predictive value of the model was significantly higher than that of CEA (0.633; 95% CI: 0.541 to 0.719), CA199 (0.567; 95% CI: 0.474 to 0.656), CA125 (0.517; 95% CI: 0.424 to 0.608) and CA724 (0.592; 95% CI: 0.499 to 0.680) (Figure 2A-2B) (Supplementary Table S3).

### Combination of the diagnostic model with clinical biomarkers

**Figure 3:**
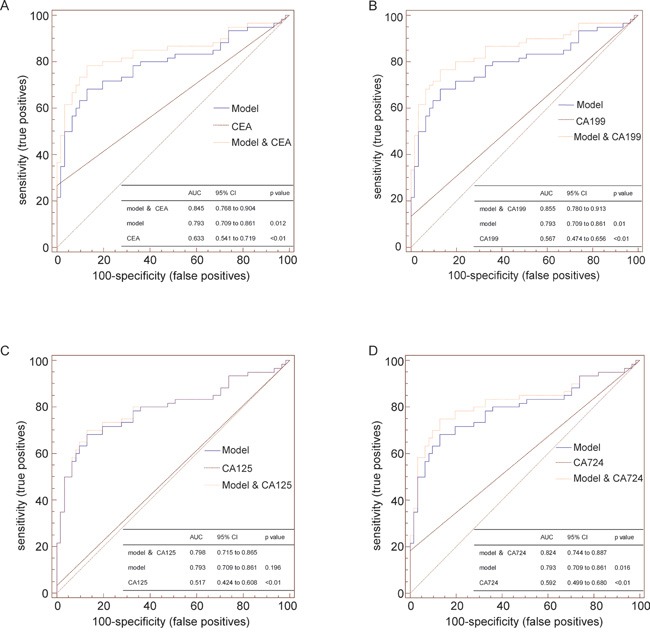
Comparison of the diagnostic abilities of the model consisting of the serum levels of three lncRNAs, conventional biomarkers, and combinations of the model with individual biomarkers The AUCs of the model and the clinical biomarkers alone were much lower than those of the combinations **A, B** and **D.**, except for the combination of the model with CA125; the predictive ability of this was equivalent to that of the model alone **C.**

As our data indicated that the diagnostic ability of the model was complementary to that of clinical biomarkers (Supplementary Table S3), we combined the diagnostic model with those clinical biomarkers. The AUCs for each combination were 0.845 (model combined with CEA), 0.855 (model combined with CA199), 0.798 (model combined with CA125) and 0.824 (model combined with CA724). The AUCs of the model and the clinical biomarkers alone were much lower than those of the combinations, except for the combination of the model with CA125; the predictive ability of this was equivalent to that of the model alone (Figure 3A-3D).

### Correlation between marker expression and operative status and clinical characteristics

**Figure 4:**
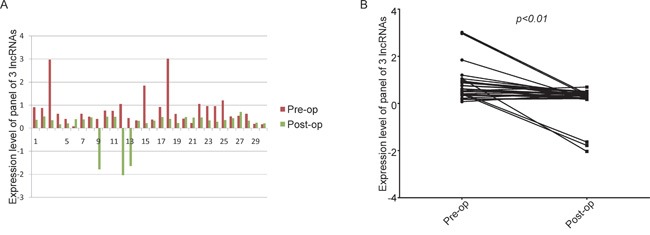
The levels of the panel of the three lncRNAs were significantly reduced after surgery A. histogram; B. line chart The expression levels of the panel of three lncRNAs in serum samples collected pre-operatively and post-operatively, as examined by real-time PCR and analyzed using the Wilcoxon signed-rank test.

We analyzed the levels of the three lncRNAs in 30 CRC patients, from whom serum samples were collected pre-operatively and post-operatively and examined by qRT-PCR. The levels of the panel of the three lncRNAs were significantly reduced after surgery (Figure 4A-4B).

We also explored whether the levels of these lncRNAs can be associated with the clinical characteristics of cancer. Therefore, we examined the association between expression of the three-lncRNA panel and clinical parameters in 60 CRC patients and found no significant association with tumor size, tumor location, TNM stage or tumor metastasis (p>0.05, Table 1).

**Table 1:** Correlations between the serum expression levels of the three-lncRNA panel and clinical parameters

Characteristics		n	p
Gender			0.761
	male	37	
	female	23	
Age			0.606
	≥60	36	
	<60	24	
Smoking status			0.372
	yes	16	
	no	44	
Drinking status			0.240
	yes	11	
	no	49	
Tumor localization			0.720
	colon	20	
	rectum	40	
pT stage			0.277
	Tis-T1	3	
	T2	8	
	T3	14	
	T4	35	
pN stage			0.199
	N0	35	
	N1	15	
	N2	10	
Metastasis			0.832
	M0	53	
	M1	7	
TNM stage			0.346
	0-I	8	
	II	24	
	III	21	
	IV	7	
Maximum tumor size, cm			0.300
	<5	23	
	≥5	34	

The relative expression levels of the three-lncRNA panel were calculated using the regression equation generated via stepwise regression analysis.

## DISCUSSION

CRC is the third most common cancer among men and the second most common cancer among women worldwide. Currently, the screening methods for CRC primarily consist of the guaiac fecal occult blood test (gFOBT), the fecal immunochemical test (FIT), flexible sigmoidoscopy (FS), colonoscopy, fecal DNA tests and computed tomographic colonography (CTC) [15]. All these methods can be less accepted by patients than blood examination, for which samples are easy to obtain, with no invasive method required and a low economic burden. However, there is no blood biomarker available to date for CRC that displays high sensitivity and specificity. In the clinic, serum concentrations of CAE, CA199, CA125 and CA724 are typically used for CRC diagnosis, but they are also used for other cancer types, such as gastric, pancreatic, and lung cancers. Additionally, the levels of these biomarkers can be elevated in patients with only digestive tract inflammation. Thus, searching for effective blood biomarkers for CRC is very important because such biomarkers would be a helpful supplement to CRC screening.

LncRNAs are reported to perform important functions in tumorigenesis and cancer development, including CRC [16–18]. Furthermore, lncRNAs have been not only studied in tumor tissue but have also been identified in the circulation in an increasing number of other cancers. For example, the lncRNA FR0348383 in urine improved diagnostic accuracy in patients undergoing prostate biopsy [19], FER1L4 was demonstrated to be present in human plasma and to be significantly down-regulated after surgery [20], and AA174084 was reported to have potential as a marker for early diagnosis of gastric cancer [21]. Although no systematic study has examined circulating lncRNAs in CRC patients, miRNAs, another class of non-coding RNA, have been widely studied in the circulation of CRC patients [22]. Thus, circulating lncRNAs may also serve as biomarkers for CRC diagnosis.

In this study, we found that 13 of the 17 examined CRC- or intestinal cancer-related lncRNAs to be detectable in both CRC patients and healthy individuals by qRT-PCR (Supplementary Table S1). Overall, three lncRNAs, LOC285194, RP11-462C24.1 and Nbla12061, were up-regulated in serum from 61 CRC patients compared with 60 healthy controls. Using these three lncRNAs, we constructed a diagnostic model to distinguish CRC patients from healthy individuals via stepwise regression. This model showed a greater ability to discriminate CRC patients from healthy individuals (AUC of 0.793 (95% CI: 0.709 to 0.861)) than any single conventional biomarker (Supplementary Table S3). The sensitivity and specificity of this model were 68.33% and 86.89%, respectively. The diagnostic abilities of combinations of this model with CEA, CA199 or CA724 were equivalent (Supplementary Figure S1), and these combinations displayed greater diagnostic ability than the combination of the model with CA125, the model alone and any single biomarker. In summary, combining the model with one biomarker resulted in higher diagnostic ability.

In addition, although the results indicated that the expression level of the three-lncRNA panel was not associated with any clinical characteristic of CRC, the level was significantly decreased in CRC patients post-operatively. This observation suggests that this panel of three lncRNAs could be used to monitor CRC development and recurrence.

The three lncRNAs constituting the diagnostic model were previously identified in CRC and gastrointestinal cancer. Indeed, the relative levels of LOC285194 have been shown to be significantly lower in CRC tissues and colorectal cancer cell lines than in adjacent normal tissues and a normal intestinal mucous cell line, respectively, and decreased expression of LOC285194 has been associated with poor prognosis [23]. A subsequent study demonstrated that LOC285194 acts as a p53-regulated tumor suppressor in colon cancer cells, partly by repressing miR-211 [24]. In addition, LOC285194 expression was found to be significantly down-regulated in esophageal squamous cell carcinoma tissues as well as osteosarcoma and pancreatic ductal adenocarcinoma tissues and cells compared with adjacent normal tissues and a normal cell line [25–27]. In addition, Nbla12061 has been found to display significantly higher expression in gastric cancer tissue than in normal gastric mucosal tissue and in serum from gastric patients than in serum from healthy controls. However, Nbla12061 was not selected as a biomarker for gastric cancer in a previous study [28]. Moreover, Nbal12061 was found to be down-regulated in neuroblastoma, in association with a poor prognosis [29]. The lncRNA RP11-462C24.1 was reported to be less abundant in cancer tissues than in adjacent normal samples from CRC patients and was demonstrated to have potential as a novel prognostic marker for CRC [30]. Considering all of these findings, we conclude that lncRNAs LOC285194 and RP11-462C24.1 function as tumor suppressors in CRC. However, both of these lncRNAs were up-regulated in serum from CRC patients in our study, inconsistencies that were previously reported in another study of circulating lncRNAs [28] and in our previous study of another disease (unpublished data).

The incongruity of variation in serum and tissue lncRNA levels between cancer and normal specimens led us to explore the source and form of circulating lncRNAs. Because lncRNAs were detectable in both patients and healthy controls, the most likely sources of circulating lncRNAs are tumor cells and normal tissue cells. The differences in lncRNA expression between the two groups may be caused by a secretory mechanism. Certain tumor suppressors, such as LOC285194 and RP11-462C24.1, were up-regulated in sera from CRC patients, which may be caused by increased secretion of these lncRNAs by tumor cells, representing a mechanism of tumorigenesis. The extrusion of tumor suppressors occurs in tumorigenesis, and simultaneous up- or down-regulation of lncRNAs in both tumor tissue and patient serum may be caused by changes in their synthesis and secretion. Regardless, lncRNA secretion may be controlled by different mechanisms and involve distinct pathways, requiring further study. To date, the presence of lncRNAs in serum has been widely studied. It has been demonstrated that lncRNAs are protected by extracellular vesicles in the circulation, enabling their stable presence in serum [31, 32]. Extracellular vesicles function as intercellular messengers by transporting molecules from cell to cell, facilitating the function of these molecules, and this process represents an intriguing strategy for treating diseases [33]. Indeed, studying circulating lncRNAs may also provide new directions for cancer treatment.

Serum lncRNAs are helpful novel biomarkers for disease diagnosis due to their easy acquisition and detection and their relationship to disease. In this study, we analyzed 17 lncRNAs in sera from CRC patients and constructed a diagnostic model using serum samples from 61 patients and 60 healthy controls. As additional lncRNAs are identified, further analysis will be performed to identify more effective biomarkers; analysis of large sample sets is needed to provide more reliable data. Moreover, serum lncRNAs hold potential for both monitoring recurrence and as novel therapeutic targets.

In conclusion, our data show that a combination of three lncRNAs in serum represents a new supplementary method for CRC screening. This achievement may improve the ability to screen for CRC in clinical practice and may provide new directions for future studies, such as the sources, functions and clinical value of circulating lncRNAs.

## MATERIALS AND METHODS

### Study design

This study included five steps.

Step I. We measured the serum levels of the 17 selected lncRNAs in 10 pre-operative CRC patients and 10 healthy individuals to determine which lncRNAs are detectable in these samples.

Step II. To identify differentially abundant lncRNAs, those lncRNAs detected in serum samples from CRC patients and healthy controls were evaluated in the sera of 30 pre-operative CRC patients and 31 healthy individuals.

Step III. Those lncRNAs displaying different levels between CRC patients and healthy individuals in step II were examined in serum samples from an additional 30 CRC patients and 30 healthy individuals.

Step IV. The data obtained in steps II and III were used to construct a diagnostic model. CEA, CA199, CA125 and CA724 levels were evaluated in the serum samples obtained in the above two steps. We compared the diagnostic value of the model and with that of each biomarker.

Step V. The levels of the lncRNAs included in the diagnostic model were evaluated in sera from 30 post-operative CRC patients whose pre-operative serum samples were used in steps II and III. We also examined correlations between the serum levels of the lncRNAs and the clinical characteristics of all 60 studied CRC patients.

### Patients and specimens

All serum samples were randomly selected from samples collected by Provincial Hospital Affiliated to Shandong University. All patients recruited had not received anticancer treatment pre-operatively and had been diagnosed with CRC based on histopathological examination. Sera were collected from the peripheral blood of all subjects using standard procedures and stored in RNase- and DNase-free tubes (Axygen, Tewksbury, MA, USA) at −80°C until extraction of total RNA.

The data obtained from all of the subjects' medical records included age, gender, alcohol consumption, smoking status, and CRC characteristics such as tumor size, tumor location, histology, and statuses of lymphatic metastasis, vascular invasion and distant metastasis. The CRC pathological stage was determined based on the tumor-node-metastasis staging system of the International Union against Cancer (5th Edition). Written informed consent was obtained from all participants. Ethical consent was granted by the Committees for Ethical Review of Research involving Human Subjects of the Provincial Hospital Affiliated to Shandong University.

### RNA isolation, reverse transcription (RT), and quantitative PCR (qPCR)

Total RNA was extracted from serum samples using a Blood Total RNA Isolation Kit (RP4001, BioTeke Corporation, Beijing, China) according to the manufacturer's protocol; the resulting extract was eluted using pre-heated (65°C) RNase-free water. RT and qPCR kits were utilized to evaluate lncRNA levels in the samples according to the manufacturer's protocol. The 20-μl RT reactions were performed using a PrimeScript™ RT Reagent Kit (Takara, Dalian, China) consisting of 10 μl total RNA solution, 1 μl PrimeScript RT Enzyme Mix I, 1 μl RT Primer Mix, 4 μl 5× PrimeScript Buffer, and 4 μl RNase-free dH_2_O. qPCR was performed using SYBR^®^ Premix Ex Taq™ (Takara, Dalian, China) by mixing 2 μl of the RT reaction products with 10 μl SYBR^®^ Premix Ex Taq II™, 0.4 μl gene-specific forward and reverse primers (10 μM), and 7.2 μl nuclease-free water. The primers used in this study are summarized in Supplementary Table S1. All reactions were performed using the Roche LightCycler 480II thermal cycler (Roche, Switzerland) with the following protocol: 95°C for 30 s followed by 45 cycles of 95°C for 5 s and 60°C for 30 s. Amplification of the appropriate products was confirmed by melting curve analysis. β-Actin mRNA was simultaneously amplified for normalization of the relative levels of lncRNA because β-actin has been demonstrated to be the most suitable reference housekeeping gene for serum lncRNA expression analysis [28]. The sequences of the primers used for β-actin were 5'-AAGCCACCCCACTTCTCTCTAA-3' (forward) and 5'-AATGCTATCACCTCCCCTGTGT-3' (reverse). The reactions were performed in duplicate or triplicate according to the acquired volume of the samples. CT values> 40 were considered negative results. The relative expression of the target lncRNAs was calculated using the 2^−ΔCT^ method.

### Serum biomarker detection

Serum levels of CEA, CA199, CA125 and CA724 were measured using the Roche Cobas 8000 system (Roche, Switzerland).

### Statistical analysis

All statistical analyses were performed using SPSS 17.0 software (SPSS Inc.). Differences in the serum levels of lncRNAs between CRC patients and healthy individuals were evaluated using a two-tailed t-test. Multivariate classification models were constructed to determine the combination of selected lncRNAs with the greatest predictive ability for cancer. Receiver operating characteristic (ROC) curves were plotted, and the area under the ROC curve (AUC) was calculated to assess the specificity and sensitivity of distinguishing CRC patients from healthy controls. ANOVA was used to examine correlations between lncRNA levels and clinical parameters when more than two groups were compared; otherwise, a t-test was used. The Wilcoxon signed-rank test was used to compare serum lncRNA levels in CRC patients between pre-operative and post-operative time points. A value of p<0.05 was considered statistically significant.

## SUPPLEMENTARY FIGURES AND TABLES


