# Supplementary material for: Long non-coding RNAs LOC285194, RP11-462C24.1 and Nbla12061 in serum provide a new approach for distinguishing patients with colorectal cancer from healthy controls
Source: Oncotarget. 2016 Sep 23;7(43):70769–78. doi: 10.18632/oncotarget.12220 (PMC5342588; doi:10.18632/oncotarget.12220)
Supplement: Supplementary file 1 [file oncotarget-07-70769-s001.pdf]

Long non-coding RNAs LOC285194, RP11-462C24.1 and Nbla12061 in serum provide a new approach for distinguishing patients with colorectal cancer from healthy controls

SUPPLEMENTARY FIGURE AND TABLES

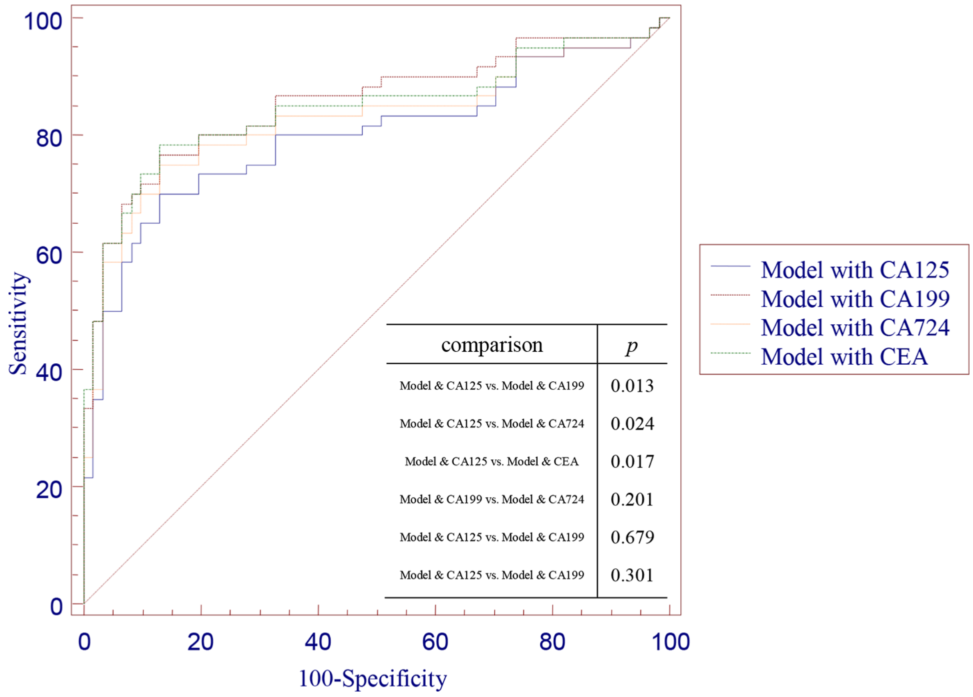

Supplementary Figure S1: The comparison of the diagnostic abilities of combinations of this model with traditional biomarkers. The diagnostic abilities of combinations of this model with CEA, CA199 or CA724 were equivalent. these combinations displayed greater diagnostic ability than the combination of the model with CA125.

Supplementary Table S1: Primer sequence list and statistical results

Gene	Primer sequence	Phase I	phase II	phase II + III
CCAT1	Forward: 5'-CATTGGGAAAGGTGCCGAGA-3'	N		
	Reverse: 5'-ACGCTTAGCCATACAGAGCC-3'			
RP11-462C24.1	Forward: 5'-GAACTTCCTCCCACTTATCCCTTAG-3'	P	0.013	0.026
	Reverse: 5'-GATGATTTTTGCTGTTCTAAGTGATGTAT-3'			
lincRNA-p21	Forward: 5'-TGTTGCATTGTTGCATCATC-3'	P	<0.01	0.384
	Reverse: 5'-TTTCTTCCAGTGGTGAGTGG-3'			
PCAT1	Forward: 5'-TGAGAAGAGAAATCTATTGGAACC-3'	N		
	Reverse: 5'-GGTTTGTC-TCCGCTGCTTTA-3'			
CCAT2	Forward: 5'-CCCTGGTCAAATTGCTTAACCT-3'	P	0.071	0.942
	Reverse: 5'-TTATTCGTCCCTCTGTTTTATGGAT-3'			
ZFAS1	Forward: 5'-ACGTGCAGACATCTACAACCT-3'	N		
	Reverse: 5'-TACTTCCAACACCCGCAT-3'			
H19	Forward: 5'-TGCTGCACTTTACAACCACTG-3'	P	0.052	
	Reverse: 5'-ATGGTGTCTTTGATGTTGGGC-3'			
HOTAIR	Forward: 5'-GGTAGAAAAAGCAACCACGAAGC-3'	P	0.138	
	Reverse: 5'-ACATAAACCTCTGTCTGTGAGTGCC-3'			
MALAT1	Forward: 5'-AACGCAGACGAAAATGGAAAGA-3'	N		
	Reverse: 5'-CCTTCTAACTTCTGCACCACCAGA-3'			
MEG3	Forward: 5'-CTGCCCATCTACACCTCACG-3'	P	0.119	
	Reverse: 5'-CTCTCCGCCGTCTGCGCTAGGGGCT-3'			
PRNCR1	Forward: 5'-CCAGGGGGAAACACACAG-3'	P	0.199	
	Reverse: 5'-AAATGGCAGTTTCCTTCAATG-3'			
LET	Forward: 5'-GTTGTTGTTGCATTGGGGT-3'	P	0.48	
	Reverse: 5'-AAGATGGAGAGTGGAGCCT-3'			
LOC285194	Forward: 5'-TGTGCCTGTTTGACCTCTGA-3'	P	<0.01	<0.01
	Reverse: 5'-AGGAAGGATAAAAGACCGACCA-3'			
PTENP1	Forward: 5'-AGTCACCTGTTAAGAAAATGAGAAGACAAA-3'	P	0.306	
	Reverse: 5'-CTGTCCCTTATCAGATACATGACTTTCAA-3'			
Nbla10727	Forward: 5'-CAGTCAGCCTCAGTTTCCAA-3'	P	0.143	
	Reverse: 5'-AGGCAGGGCTGTGCTGAT-3'			
Nbla12061	Forward: 5'-ATGTTAGCTCCCAGCGATGC-3'	P	<0.01	<0.01
	Reverse: 5'-CTAACTGCCAAAAGGTTTTCC-3'			
ATB	Forward: 5'-TCTGGCTGAGGCTGGTTGAC-3'	P	0.024	0.082
	Reverse: 5'-ATCTCTGGGTGCTGGTGAAGG-3'			

N: negative
P: positive

Supplementary Table S2: Multivariate stepwise logistic regression analysis* of the three lncRNAs in the sera of patients with colorectal cancer

lncRNA	Regression coefficient	p
LOC285194	14.596	<0.001
RP11-462C24.1	23.376	<0.001
Nbla12061	-2.515	0.04

*Stepwise selection was used to determine the diagnostic values of serum lncRNAs for colorectal cancer (CRC). The combination of LOC285194, RP11-462C24.1 and Nbla12061 was chosen as the strongest panel for HCC diagnosis.
The regression equation: The relative expression level of a panel of three lncRNAs = -0.02+14.596×LOC285194+23.376×RP11-462C24.1-2.515×Nbla12061.

Supplementary Table S3: Diagnostic ability of the model and conventional biomarkers and the comparison of the model to the markers

	AUC	95%CI	sensitivity	specificity	p
Model	0.793	0.709 to 0.861	68.33	86.89	
CEA	0.633	0.541 to 0.719	26.67	100	0.013
CA199	0.567	0.474 to 0.656	13.33	100	<0.01
CA125	0.517	0.424 to 0.608	3.33	100	<0.01
CA724	0.592	0.499 to 0.680	18.33	100	<0.01

The area under the curve (AUC, from the receiver operating characteristic curves) is shown together sensitivity and specificity. A value of $P<0.05$ was considered the comparison of the diagnostic of the model with other set statistically significant.
CI: confidence interval.
